# Quantitating and Dating Recent Gene Flow between European and East Asian Populations

**DOI:** 10.1038/srep09500

**Published:** 2015-04-02

**Authors:** Pengfei Qin, Ying Zhou, Haiyi Lou, Dongsheng Lu, Xiong Yang, Yuchen Wang, Li Jin, Yeun-Jun Chung, Shuhua Xu

**Affiliations:** 1 Chinese Academy of Sciences (CAS) Key Laboratory of Computational Biology, Max-Planck Independent Research Group on Population Genomics, CAS-MPG Partner Institute for Computational Biology, Shanghai Institutes for Biological Sciences, Chinese Academy of Sciences, Shanghai 200031, China; 2 Ministry of Education (MOE) Key Laboratory of Contemporary Anthropology, School of Life Sciences and Institutes of Biomedical Sciences, Fudan University, Shanghai 200433, China; 3 Integrated Research Center for Genome Polymorphism, Department of Microbiology, The Catholic University Medical College, Socho-gu Seoul 137-701, Korea; 4 School of Life Science and Technology, ShanghaiTec University, Shanghai 200031, China; 5 Collaborative Innovation Center of Genetics and Development, Shanghai 200438, China

## Abstract

Historical records indicate that extensive cultural, commercial and technological interaction occurred between European and Asian populations. What have been the biological consequences of these contacts in terms of gene flow? We systematically estimated gene flow between Eurasian groups using genome-wide polymorphisms from 34 populations representing Europeans, East Asians, and Central/South Asians. We identified recent gene flow between Europeans and Asians in most populations we studied, including East Asians and Northwestern Europeans, which are normally considered to be non-admixed populations. In addition we quantitatively estimated the extent of this gene flow using two statistical approaches, and dated admixture events based on admixture linkage disequilibrium. Our results indicate that most genetic admixtures occurred between 2,400 and 310 years ago and show the admixture proportions to be highly correlated with geographic locations, with the highest admixture proportions observed in Central Asia and the lowest in East Asia and Northwestern Europe. Interestingly, we observed a North-to-South decline of European gene flow in East Asians, suggesting a northern path of European gene flow diffusing into East Asian populations. Our findings contribute to an improved understanding of the history of human migration and the evolutionary mechanisms that have shaped the genetic structure of populations in Eurasia.

The interaction between European and Asian populations has been historically influenced by many factors, including the political conditions created by great empires such as the Roman (31 BC–250 AD) and Mongolian empires (1207 AD–1360 AD) that fostered communication between European and Asian populations, and the famous Silk Road (206 BC) which acted historically as a commercial, religious, and cultural network interlinking the trade routes across the Eurasian landmass that connected East Asia with the Mediterranean and Europe. This raises the question of whether genetic admixture occurred during the interaction between people from different regions, particularly between Europe and East Asia. If there has been gene flow between populations, we can, in principle, estimate the time and magnitude of this gene flow by analyzing genomic data from modern human populations. Previous studies have revealed that the populations of Central Asia, such as Uygur, are typically admixed with genetic contributions from both European and East Asian populations^1^,^2^,^3^,^4^,^5^,^6^,^7^. However, only a few cases of genetic admixture among other Eurasians, particularly between East Asians and Northwestern Europeans have been reported^8^,^9^. These studies reported the Asian or American contribution in European populations. But the European gene flow in Asians has not been well studied yet.

Here we present what is, to the best of our knowledge, a systematic investigation of genetic admixture between different European and Asian populations using genome-wide single nucleotide polymorphism (SNP) data from 34 populations, including seven European groups (EUR), 9 Central or South Asian groups (CSA), and 17 East Asian groups (EAS) and one Tibetan group from Tibet Plateau. Based on statistical analysis using 4 Population Test (f_4_-test) and 3 Population Test (f_3_-test)^10^,^11^, we detected gene flow between Europeans and Asians were prevalent for most populations including even those from west-northern Europe and East Asia which have been generally regarded as less admixed. We quantitatively estimated the magnitude of these gene flows using two different f_4_ statistical approaches: F_4_ Ratio Estimation^12^, and Regression Ancestry Estimation^10^. And finally, we estimated the time of admixture events based on their admixture linkage disequilibrium (ALD) using *ROLLOFF* software^12^.

## Results

### Evidence of Gene Flow between EUR and EAS

**Figure 1:**
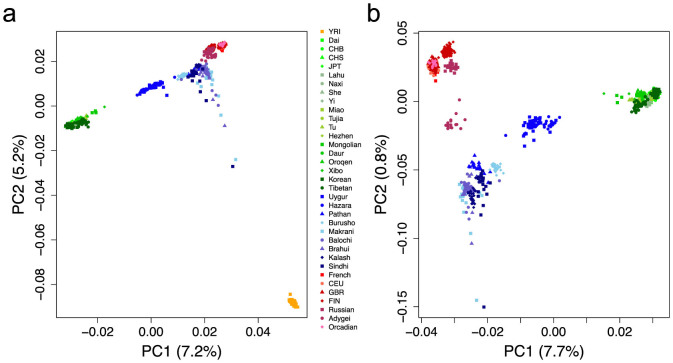
PC plots indicate potential gene flow between EUR and EAS. (a) PC plot of 1,219 samples from 35 populations that were clustered into EAS, EUR, CSA, and African. (b) Fine resolution of the PC plot after removing YRI. Both plots were based on 96,538 pruned SNPs to reduce linkage disequilibrium relationships.

We obtained genome-wide data of 34 Eurasian populations from 1000 Genomes Project (1 KG)^13^, the Human Genome Diversity Panel (HGDP)^14^ and other studies^1^,^15^. In total, 1,132 Eurasian samples with 186,506 SNPs integrated from different technical platforms were used for analysis (see Methods). To study the signal of gene flow between West and East Eurasian populations (European and Asian populations in Table 1), firstly we performed Principal Component Analysis (PCA)^16^ both with and without respectively African populations based on 96,538 random SNPs avoiding high linkage disequilibrium. We observed that some clusters of West and East Eurasians are slightly shifted towards each other, indicating potential admixture between them (Fig. 1). There is a clear ‘cline’ between West and East clusters. These shifts of PC patterns are especially obvious for most CSA, some northern EAS including Mongolian, Xibo, Hezhen, Oroqen etc., and eastern EUR including Russian, Finnish and Adygei. We then initiated formal testing for the presence of gene flow.

**Figure 2:**
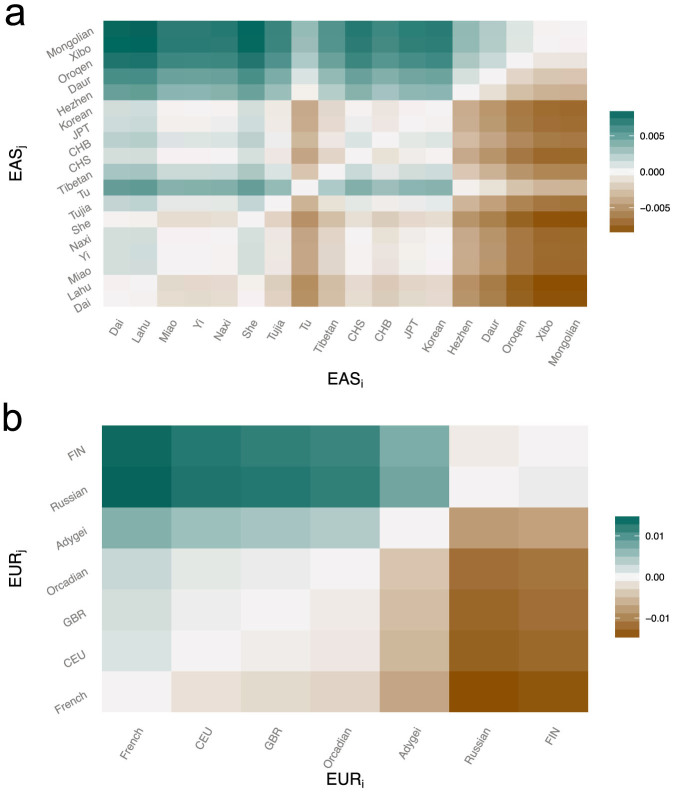
Determining surrogates of ancestral populations. *f*_4_(*YRI*, EUR; *EAS_i_*, *EAS_j_*) and *f*_4_(*YRI*, EAS; *EUR_i_*, *EUR_j_*) were used to identify least-admixed populations as ancestral surrogates of (a) EAS and (b) EUR, respectively. Positive values in (a) indicate there is less EUR ancestry in EAS_i_ than in EAS_j_, and positive values in (b) indicate there is less EAS ancestry in EUR_i_ than in EUR_j_.

We assessed the gene flow between EUR and EAS using f_4_-test and f_3_-test for each Eurasian group. The f_4_-test and f_3_-test, which are model-based statistics, were designed to measure genetic drift along lineages quantitatively to detect gene flow between populations^10^,^11^. In the current study, our samples did not cover all European regions. For example, Southern European populations were not included in the analysis because they may have been affected by a recent genetic contribution from populations with African ancestry. Several studies have revealed evidence of sub-Saharan African admixture among southern European populations but only rarely among northern European populations^12^,^17^,^18^. Since ancestral populations predating admixture are unavailable, an alternative is to determine proper surrogates of ancestral EUR and EAS. We have employed an approach based on f statistics for each pair of EAS, EAS_i_ and EAS_j_, to compare the quantitative contribution they inherited from EUR (see Methods). We can therefore choose the least-admixed EAS as a surrogate of ancestral EAS. As a result, based on phylogeny as Supplementary Fig. S1, Dai from southern China could be considered to be a surrogate of ancestral EAS (Fig. 2a). Similarly, we applied our approach for each pair of EUR and found French holds the less genetic contribution from EAS than other EUR, and therefore can be considered as a surrogate of ancestral EUR (Fig. 2b). We assumed a Hybrid Isolation (HI) model for admixed Eurasian populations with ancestries from both EUR and EAS, as shown in Supplementary Fig. S1. For each group of EAS, we assessed whether the statistic *f*_4_(*YRI*, *French; Dai, EAS_i_*), significantly deviates from zero (see Methods). If so, it should indicate gene flow from EUR. Otherwise, the population should have entire EAS ancestry. Accordingly, we found that most EAS significantly deviated from the (*YRI*(*French, (Dai, EAS_i_*))) topology (Z-score>2) (Table 1 and Supplementary Table S1), indicating gene flow from EUR. We similarly tested EAS gene flow in each group of EUR by test *f*_4_(*YRI*, *Dai; French, EUR_i_*) (see Methods). Most EUR significantly violated the (*YRI*, ((*French, EUR_i_),Dai*) topology (Z-score>2) (Table 1 and Supplementary Table S1), indicating that most EUR in our study inherited a genetic contribution from EAS ancestry.

For CSA, we used two f_4_-tests, *f*_4_(*YRI*, *French, Dai; CSA_i_*) and *f*_4_(*YRI*, *Dai; French, CSA_i_*), and one f_3_-test *f*_3_(*CSA_i_, French, Dai*). Most CSA showed an extremely significant violation of the (*YRI*, (*French,* (*Dai, CSA_i_*))) topology (Table 1 and Supplementary Table S1), which indicates that these CSA (such as Uygur, Haraza, Pathan, Burusho, Kalash and Sindhi) are typical admixed populations with a high level of gene flow from both EUR and EAS ancestries. Different from other CSA, Makrani showed significant signal of African ancestry. And there was no admixture signal for Brahui.

### Quantitative Estimation of Gene Flow

To estimate the genetic contributions of EUR and EAS ancestries, we first performed a linear regression procedure^10^, which assumes that mixed populations share similar demographical histories. One Oceania population Papuan, which were not considered experiencing any recent admixture with EUR or EAS, was added into our model as an out-group^12^. For EAS with phylogeny displayed in Supplementary Fig. S2, we plotted *f*_4_(*YRI*, *French, Dai, EAS_i_*) against *f*_4_(*YRI*, *Papuan*; *French, EAS_i_*), and each group should fall along a line with a negative slope (equation (3)) if most groups shared same degree of drift T and W. By carrying out the least-squares that best fit all EAS data, we could estimate the parameters of the linear model, allowing us to calculate the admixture proportion for each group. As expected, we observed a well-fit linear model for all EAS in our study, with a correlation coefficient *r*^2^ = 0.8 (Supplementary Fig. S3a), which indicates that most EAS are likely to have inherited alleles from the same ancestral populations and shared similar demographical histories (same T and W in Supplementary Fig. S2). We subsequently calculated the admixture proportion given the values of drift T and W that could be estimated based on parameters of the linear model. An estimation of admixture proportions for each EAS is provided in Table 2. We detected 2.8 ± 0.2% EUR ancestry in Northern Han Chinese (CHB), which was more than that in Southern Han Chinese (CHS; 1.7 ± 0.1%), Japanese (2.2 ± 0.2%) and Korean (1.6 ± 0.2%) populations. Northeast Asians such as Oroqen, Mongolian, Hezhen, and Daur (nomads who historically lived alongside Russians and Caucasians) inherited significantly more alleles from EUR: Mongolian 10.9 ± 0.1%, Oroqen 9.6 ± 0.2%, Daur 8.0 ± 0.2%, and Hezhen 6.8 ± 0.2%.

Similarly, we plotted *f*_4_(*YRI*, *Dai; French, EUR_i_*) against *f*_4_(*YRI*, *Papuan*; *EUR_i_, Dai*) for EUR, in which a linear model provided a good fit to the data (*r*^2^ = 0.9) (Supplementary Fig. S3b), indicating most groups underwent the same amount of drift as measured by W and M (Supplementary Fig. S2). The proportions of admixture from ancestral EUR and EAS were estimated, and are shown in Table 2. CEU populations mostly originating from France and Germany had a small fraction (0.7 ± 0.8%) of genetic material from EAS. People from Great Britain such as British (GBR) and Orcadian inherited 2.5%–3.8% from ancestral EAS. Finnish (FIN) and Russians inherited significantly more genetic material (>12%) from ancestral EAS, which is consistent with their historical record of admixture with Mongolian populations. Besides, Adygei from Caucasus inherited 3.2 ± 1.0% from ancestral EAS.

This regression method was unsuitable for studying CSA, because there was no correlation between *f*_4_(*YRI*, *Dai; French,* *CSA_i_*) against *f*_4_(*YRI*, *Papuan; Dai* *CSA_i_*) which is required in this approach. We therefore performed F_4_ Ratio Estimation^12^ to calculate the admixture proportion for CSA. For each CSA (CSA_i_), the admixture proportion could be estimated directly from the ratio of these two f_4_-tests *f*_4_(*YRI*, *Papuan; CSA_i_,* *Dai*) and *f*_4_(*YRI*, *Papuan; French,* *Dai*) (equation (4)). The results (Table 2) revealed that CSA, located in the middle of the Eurasian continent, mostly are typical admixed populations with a high level of gene flow from EUR and EAS ancestries. Uygur from Northwest China exhibit 52.4% EUR ancestry. For those populations from Pakistan, Pathan exhibits 78.4% EUR ancestry. Burusho and Hazara have 67.9% and 50.2% EUR ancestry, respectively. Admixture proportions for some CSA are relatively small, for which Kalash has 20.7% EUR ancestry, Sindhi 11%, Balochi 2.1%.

We also performed F_4_ Ratio Estimation to estimate admixture proportion for EAS and EUR. Using this method, we obtained comparable results to the regression method (Supplementary Table S2). For example, we detected 9.7% EUR ancestry in the Mongolian population. EUR ancestry is more pronounced among Northern Han Chinese than southern Han Chinese. In addition, Russian and Finnish populations show ~13% Asian ancestry.

### Estimation of Admixture Times

To provide a more detailed description of demographical history, we attempted to estimate the time that admixture events occurred. Given the low level of gene flow for some Eurasian groups, we needed an accurate and sensitive method to estimate admixture times. We used *ROLLOFF*, which examines pairs of SNPs and assesses how ALD decreases with genetic distance in admixed populations. We examined each admixed EAS using *ROLLOFF* with EAS (Dai) and EUR (French) ancestries. By fitting an exponential distribution to each run of *ROLLOFF* with least-squares, we obtained the number of generations since admixture. As shown in Table 2, Han Chinese (CHB) received recent genetic input from EUR approximately 48 ± 1.2 generations (1383 years) ago assuming a generation time of 29 years^19^. Similarly, Korean populations received recent gene flow from EUR approximately 45 ± 3.9 generations ago. EUR gene flow reached Japanese populations 60.8 ± 31.2 generations (1,763 years) ago, which probably followed the continental migration of Yayoi that began ~2300 years ago and continued for the next 1000 years^20^. The admixture of EUR in Xibo population collected in Xinjiang could be dated back to 10.7 ± 0.7 generations, or ~310 years ago. In the past, Xibo populations lived in Siberia and North-Eastern China. In the mid-18^th^ century, part of the Xibo population migrated to North-Western China due to the policy of guarding the frontier by the government of the Qing Dynasty. This estimation of admixture time correlates accurately with this historical event. Interestingly, according to our estimation, Mongolians received their European ancestry 32.9 ± 0.9 generations, or 954 years ago. This is around the time of the expansion of Mongolians in military and politics in the 12^th^ century, who in turn built the largest contiguous land empire in human history. However, we did not always observe the extent of ALD with genetic distance due to the extremely low level of gene flow for some groups, such as CEU and CHS.

Similar approaches were applied for both EUR and CSA. Two populations from Great Britain (GBR and Orcadian) shared a similar recent admixture time with EAS (82.8 ± 39.6 and 78.1 ± 8.6 generations, respectively), which were older than other EUR. Adygei received their EAS ancestry 24.1 ± 1.2 generations, or 699 years ago. The gene flow from EAS to Finnish and Russian populations could be dated back 64.2 ± 1.1 and 45.2 ± 1.3 generations, or 1,862 and 1,311 years, respectively. Our estimation of admixture between EAS and EUR in CSA populations was 26.4 ± 0.5 generations for Uygur, 24.4 ± 0.2 generations for Hazara, and 49.2 ± 1.1 and 51.2 ± 2.3 generations for Burusho and Pathan, respectively. The small fraction of EUR ancestry in Kalash was derived from 61.8 ± 5.3 generations ago. It's 70.7 ± 3.1 for Sindhi and 82 ± 4.1 for Balochi, respectively. However, we were aware of the complex demographic history of CSA, and multiple waves of admixture may have occurred at different times. Based on the decay of the extended ALD pattern, which is likely to be affected by bottlenecks and multiple or continuous gene flows, the estimation of *ROLLOFF* tends to correspond reasonably well with more recent admixture events^12^.

### Admixture in the Uygur

According to the historical records, ancestors of Uygur can be traced to ancient Chidi and Dingling populations living in this region in the 3^rd^ century B.C. The Silk Road crossed Xinjiang which improved the communication between EUR and EAS can be traced back to 206 B.C. Admixture is likely to have taken place since then, or even earlier. However, our study and one previous study^11^ gave a very short estimation of about 26 generations (Table 2) since admixture based on approach that assesses the decay of ALD which tends to reflect more recent events if multiple waves of admixture happened.

We next explored possible scenarios with respect to multiple admixture of the Uygur using simulation. We simulated two waves of admixture with the earlier one occurred around 110 generations ago and the later one occurred around 25 generations ago which represents a recent admixture event. Admixture time based on HI model was estimated by two approaches, one was based on number of ancestral segments (equation (5)), while the other was based on assessing the decay of ALD. According to the results of simulated data (Supplementary Fig. S4 and Supplementary Table S3), it was apparent that the second approach through assessing the decay of ALD tended to capture information of the more recent admixture event, and it was even more pronounced in those scenarios with higher weight of the second admixture.

In empirical data, which consists 42 Uygur samples genotyped by Affymetrix Genome-Wide Human SNP Array 6.0 (Affy6.0), we estimated admixture time by inferring recombination on Uygur chromosomes with HAPMIX^21^. Expected admixture time was estimated to be 54 generations by the average number of segments (equation (5)), which is much larger than the estimation of 26 generations using ALD information (Supplementary Table S3). This estimation could be an intermediate of ancient admixture and recent admixture. As such, our results indicated the admixture in Uygur is compatible with multiple waves of admixture between EAS and EUR.

### A Northern Path of EUR Gene Flow Diffusing into EAS

**Figure 3:**
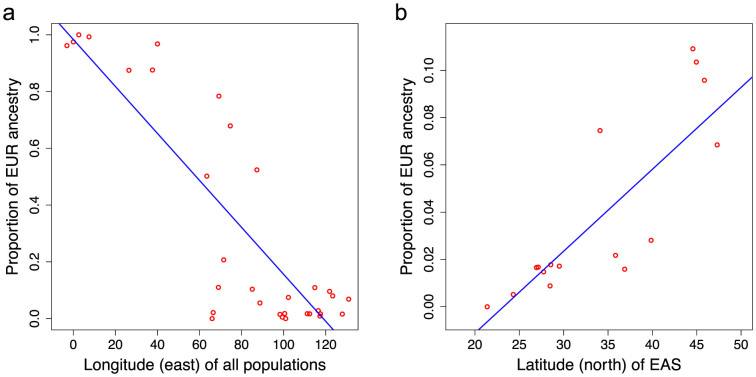
Correlation of gene flow and geographical locations of populations. (a) West-to-East descent of EUR ancestry in 33 populations (except for Makrani who significantly admixed with Africans) (*r*^2^ = 0.74). (b) North-to-South descent of EUR ancestry in 17 populations of EAS (*r*^2^ = 0.71).

Apparently gene flow between EUR and EAS is highly correlated with geographical locations of populations from West to East in the Eurasian continent, as revealed by our estimations (*r*^2^ = 0.74) (Fig. 3a). More interestingly, we observed a North-to-South decline of EUR gene flow in all EAS populations (*r*^2^ = 0.71) (Fig. 3b), which suggested a northern route for EUR diffusion into EAS. Populations from the Northern part of East Asia were more likely to have genetic communication with EUR, as Silk Road facilitated the communication of East and West world^4^,^22^ and nomadic populations in Northern Asia such as Mongolian were likely to contact with people from Caucasus region or other European-like populations around them in history and intermediate the EUR gene flow into other EAS^23^. Our data reveal that populations in the North such as Mongolian, Xibo, Oroqen, Hezhen, Northern Han Chinese, and Tu inherited much more European ancestry than populations in the South, such as She, Tujia, Yi, Miao, and Southern Han Chinese. Exceptionally, Japanese and Korean populations did not receive as much gene flow from EUR (1.6% for Korean, 2.2% for Japanese) as other Northern EAS did, which is likely due to their isolated location in Northeast Asia.

## Discussion

In this study, by analyzing genome-wide SNP data, we revealed that recent genetic admixture did occur and have been prevalent in Eurasia continent, notably, gene flows have been detected even between northern European and East Asian populations which are geographically far away from each other and generally considered as well-differentiated populations.

We would like to point out that the EAS gene flow to EUR we observed in this study might not exactly came from EAS, instead, it could come from some EAS-related people who no longer live in East Asia. Similar situation could be applied to EUR. Even though Dai and French were not necessarily the true ancestors who directly contribute genetic materials to other Eurasians, using them as surrogates of ancestors would not have significantly affected the estimations of admixture. This could be observed in our simulation study for estimating admixture proportion using sister groups instead of real ancestors and with consideration of ascertainment bias. We simulated ascertainment bias by using markers with minor allele frequency (of all populations, EUR only and EAS only)>5% (Supplementary Fig. S5 and S6). The previous application of *ROLLOFF* in time estimations has also been shown to be robust using surrogates of real ancestors in previous study^11^. Our approach and simplified model in admixture estimations will still be robust when the admixture history is complicated, such as multiple admixture event or pre-mixed ancestors. The estimation of admixture proportion would not be remarkable affected (simulations in Supplementary Fig. S7 and S8), while dating admixture mainly reflects the recent admixture event (simulations in Supplementary Fig. S4 and S9,Supplementary Table S3).

In addition, our results are consistent with some recent studies^8^,^9^, while we identified and estimated EUR admixture in EAS which was not well described in previous studies. We found signals of gene flow in many populations with reasonable interpretations, especially with respect to EUR admixture in EAS.

Our estimation of admixture time was based on the assumption of an HI model, which is a simplified model for the complex untraceable admixture reality. In realistic scenarios, most admixtures between populations could be continuous or multiple waved. The real parameters of admixture such as proportions and time are usually incomprehensible. Even though some estimations here might be indecipherable to real history, we could estimate, by modeling admixture based on genetic diversity, LD and ancestral segmental distribution, many useful parameters such as the effective admixture proportion and effective admixture time which are helpful to further evolutionary and medical studies. The algorithm (*ROLLOFF*) we applied to date admixture in our study is LD-based, one tricky issue for such algorithm is that we could not precisely distinguish admixture LD from the background LD, especially when the admixture level is extremely low. That's why *ROLLOFF* lose power for dating admixture in some populations with low level of gene flow.

Taken together, although fine-scale dissection of the demographic history of human populations in Eurasia and precise estimation of evolutionary parameters need improved methods and data, our current study provided an overall picture of subsequent genetic interaction among well-differentiated populations. Our results advanced our understanding of the history of human migration and the evolutionary mechanisms that have shaped the genetic structure of populations in Eurasia.

## Methods

### Population Samples and Data

Samples of 1,256 individuals in total from 34 Eurasian populations, one African population Yoruban (YRI) and one Oceanian population Papuan were obtained in this study. Genome-wide SNP data were acquired from both public datasets (1000 Genomes Project (1 KG)^13^, the Human Genome Diversity Panel (HGDP)^14^) and other studies in which there are 100 Korean samples (South Korea), 46 Tibetans (Tibet^15^) and 44 Uygurs (Xinjiang, China^1^), genotyped by Affy6.0. Details of samples were listed in Table 1.The combined data was further filtered to exclude individuals with>10% missing genotypes, and SNPs with missing rate>10% as well as those exhibiting Hardy-Weinberg disequilibrium (p <0.001). Pairwise kinship coefficients were estimated in each population^24^. According to the relationship inference criteria, individuals in the first (e.g. full-sibs), second (e.g. half-sibs) and third (e.g. first cousins) degrees of relationship were removed from our study. At last, we obtained 1,132 Eurasian samples with 186,506 SNPs after integration and quality control.

### Principle component analysis

Principle component analysis (PCA) was performed with EIGENSOFT^16^ version 5.0.1 based on 96,538 pruned SNPs, which were randomly selected from 186,506 autosomal SNPs of all merged samples with interval distance larger than 10 kb to avoid high linkage disequilibrium.

### Modeling Admixture between EUR and EAS

We assumed that admixture events between EUR and EAS occurred following the HI model (Supplementary Fig. S1). Since ancestral populations predating admixture are unavailable, an alternative is to determine proper surrogates of ancestral EUR and EAS. An approach was employed to select the least-admixed EAS and EUR exhibiting minimal gene flow. To choose proper surrogates for ancestral EAS, we applied a set of f_4_-tests to the proposed relationship (*YRI*, (*EUR*, (*EAS_i_*, *EAS_j_*))) for each pair of EAS (EAS_i_ and EAS_j_) under consideration. 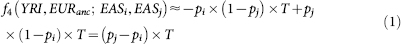
where EUR_anc_ represents the ancestral populations of EUR, p_i_ and p_j_ is the EUR contribution to EAS_i_ and EAS_j_, respectively, T is the quantity of drift depicted in Supplementary Fig. S1. Equation (1), (2), (3) and (4) were derived according to the explanation of f statistics^10^,^11^. The expectation of f statistics is mathematically appropriate to the drift paths in the admixture graph. A positive value means that EUR contribution to EAS_i_ is smaller than to EAS_j._ We can therefore easily choose the least-admixed EAS as a surrogate of ancestral EAS. For EUR, we applied similar f_4_-tests to the proposed relationship (*YRI*, ((*EUR_i_*, *EUR_j_*), *EAS*)) for each pair of EUR (EUR_i_ and EUR_j_). 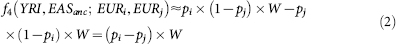
where EAS_anc_ represents the ancestral populations of EAS, p_i_ and p_j_ is the EUR_anc_ contribution to EUR_i_ and EUR_j_, respectively, and W is the quantity of drift depicted in Supplementary Fig. S1. A population consistently exhibiting positive values of the f_4_-test was treated as the surrogate of the ancestral EUR.

### Statistical Analysis to Detect Gene Flow between EUR and EAS

We applied two model-based statistics (the f_4_-test and the f_3_-test^10^,^11^) to detect gene flow between Eurasian populations. Weighted Block Jackknife^25^,^26^, which drops 5 centimorgan (cM) blocks^10^ of the genome in each run, was used to compute a standard error of the statistic. We tested for admixture by assessing whether the statistic is more than 2 standard deviations from zero.

For EAS, we assessed whether the statistics *f*_4_(*YRI*, *EUR_anc_*; *EAS_anc_*, *EAS_i_*), whose expected value should be proportional to *p_i_* × T, were significantly different from zero (Supplementary Fig. S1). A Z-score deviating from zero (≥2) could be considered as evidence of gene flow from EUR. Similarly, we examined the value of *f*_4_(*YRI*, *EAS_anc_*; *EUR_anc_*, *EUR_i_)* for EUR, which is expected to equal the product of the EAS contributions and their shared drift (1 − *p_i_*) × *W* for any EUR (Supplementary Fig. S1). In this case, a Z-score ≥2 could be considered as evidence of gene flow from EAS. In contrast to EAS and EUR, for each CSA (CSA_i_), we used two f_4_-tests, i.e. *f*_4_(*YRI*, *EUR_anc_*; *EAS_anc_*, *CSA_i_*) and *f*_4_(*YRI*, *EAS_anc_*; *EUR_anc_*, *CSA_i_*), and one f_3_-test, i.e. *f*_3_(*CSA_i_, EUR_anc_*, *EAS_anc_*). Z-scores deviating from zero provided evidence of admixture.

### Quantitative Estimation of the Gene Flow between EUR and EAS

We used two different f_4_ based approaches, Regression Ancestry Estimation^10^ and F_4_ Ratio Estimation^11^,^12^, to estimate the extent of gene flow between EUR and EAS.

### Regression Ancestry Estimation

We investigated a linear regression model based on two sets of f_4_-test values. Taking EAS as an example, the expected value of *f*_4_(*YRI*, *EUR_anc_*; *EAS_anc_*, *EAS_i_*) is proportional to *p_i_* × T. We designed an additional f_4_-test, i.e.*f*_4_(*YRI*, *Papuan; EUR_anc_*, *EAS_i_*), and the expected value is proportional to (1 − *p_i_*) × *W,* where W is a measurement of genetic drift, as described in Supplementary Fig. S2. Notably, if all EAS were derived from the same admixture event, genetic drift as measured by T or W should be similar for all these groups. For any pair of EAS, there should be:
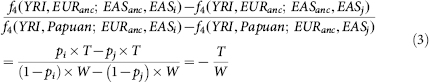
When we plotted values of *f*_4_(*YRI*, *EUR_anc_*; *EAS_anc_*, *EAS_i_*) against values of *f*_4_(*YRI*, *Papuan*; *EUR_anc_*, *EAS_i_*), all groups should be represented by a linear model with a negative slope. By carrying out a least-squares fit for all EAS groups, we extrapolated the x- and y-intercepts, which correspond to the f_4_ values expected for groups with entire EUR and EAS ancestries, to estimate the drift as measured by T and W. We could in turn estimate the admixture proportion for each group. We implemented Weighted Block Jackknife, where one chromosome was excluded each run, and studied the fluctuation of the statistic over 22 runs. The statistics estimated each run were weighted by the SNP count of the excluded chromosome.

### F_4_ Ratio Estimation

We assumed the population relationship (*YRI*, (*EUR_anc_*, ((*EAS_i_*, *EAS_anc_*), *Papuan*))) depicted in Supplementary Fig. S2. Two different f_4_-tests were used to directly estimate the proportion of admixture. The expected value of *f*_4_(*YRI*, *Papuan*; *EAS_i,_ EAS_anc_*) is proportional to *p_i_* × *W* whereas the expected value of *f*_4_(*YRI*, *Papuan*; *EUR_anc_, EAS_anc_*) is proportional to the magnitude of drift W. The ratio of these two f_4_-tests is therefore expected to be p_i_:
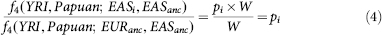
With the above procedure, we could estimate the admixture proportions of EUR and EAS for each group. The quantity of this ratio was summed over all markers, and standard error was calculated using Weighted Block Jackknife in which we dropped a block of 5 cM each repeat.

### Dating Admixture

To estimate the time of admixture events, we applied an ALD-based method implemented in *ROLLOFF*^11^,^12^, which computes the time since admixture using the rate of exponential decline of ALD. *ROLLOFF* computes the correlation between a pair of markers and a weight that reflects their allele frequency differentiation in the ancestral populations. By examining the change in this correlation with increasing genetic distance among these markers and fitting an exponential distribution to the decay of correlation by least-squares, we obtained an estimation of the date that gene flow occurred. *ROLLOFF* was also used to compute an approximately normally distributed standard error by carrying out Weighted Block Jackknife analysis, where one chromosome was excluded each run. By examining the fluctuation of the statistic, we could assess the stability of the estimation.

Ancestral origin of chromosomal segments is also informative for dating admixture. Since ancestral segments of chromosomes from mixed population could be inferred by some algorithms such as HAPMIX^21^, expected admixture time (T) could be estimated with equation
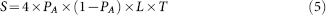
Where S is the number of segments with one allele from ancestry A and the other allele from ancestry B (as shown in Supplementary Fig. S10), P_A_ is contribution from ancestry A, L is chromosome length in Morgons and T is time in generations since admixture.

### Simulations

Both backward time and forward time simulations were conducted in this study for different purposes.

To evaluate the potential influence on our results of ascertainment bias, using surrogates instead of real ancestries and pre-mixed ancestries in the estimations of admixture, we performed coalescent simulations implemented in *ms*^27^. We used the similar parameters of demographic history as those in a previous study^11^. Simulation study of each scenario was repeated one hundred times. Command lines were listed in Supplementary Fig. S5 and Supplementary Fig. S7.

The influence of ascertainment bias was evaluated using SNPs from three different ascertains, which are based on minor allele frequency (MAF) of all simulated populations, EUR only and EAS only. We compared the estimations using all alleles and common alleles only (MAF>5%).

Forward time simulations were conducted to generate Eurasian admixed (Uygur-like) haplotypes with ancestries from ancestral surrogates (e.g. CEU and CHB), with genotype data downloaded from HapMap 3^28^ and phased by Beagle v3.3.2^29^. Ancestral segments with recombination breakpoints information in Uygur genomes were recorded during simulations. Simulated admixture scenarios were listed in Supplementary Table S3. Forward time simulations were also conducted to evaluate the influence of pre-mixed ancestries on estimation of admixture time (Supplementary Fig. S9).

## Author Contributions

S.X. conceived and designed the study. L.J. and Y.C. contributed to population samples and genotyping data. P.Q. performed data analysis, with contribution from Y.Z., H.L., D.L., X.Y. and Y.W. S.X. and P.Q. wrote the paper.

## Supplementary Material

Supplementary Information Supplementary information

## Figures and Tables

**Table 1:** Testing for gene flow between EUR and EAS

Population	Samples (after QC)	Region	Latitude | Longitude	f_4__A	f_4__B	f_3_
Dai	10 (10)	East Asia	21.4N | 101.0E	-	-	-
CHB	97 (97)	East Asia	39.9N | 116.5E	**3.7**	-	-
CHS	100 (92)	East Asia	26.9N | 117.5E	**2.3**	-	-
JPT	89 (89)	East Asia	35.9N | 138.6E	**2.6**	-	-
Lahu	10 (10)	East Asia	24.3N | 99.4E	0.5	-	-
Naxi	10 (9)	East Asia	27.7N | 98.3E	1.4	-	-
She	10 (9)	East Asia	28.5N | 117.3E	0.9	-	-
Yi	10 (10)	East Asia	28.6N | 100.5E	1.8	-	-
Miao	10 (10)	East Asia	27.1N | 112.4E	1.9	-	-
Tujia	10 (10)	East Asia	29.5N | 111.2E	1.8	-	-
Tu	10 (10)	East Asia	34.1N | 102.3E	**7.6**	-	-
Hezhen	10 (10)	East Asia	47.3N | 131.0E	**6.2**	-	-
Mongolian	10 (10)	East Asia	44.6N | 114.9E	**10.3**	-	-
Daur	10 (10)	East Asia	51.6N | 123.4E	**7.3**	-	-
Oroqen	10 (10)	East Asia	45.9N | 121.9E	**8.6**	-	-
Xibo	9 (9)	East Asia	45.0N | 85.1E	**9.85**	-	-
Korean	100 (100)	East Asia	36.9N | 127.9E	**2.55**	-	-
Tibetan	46 (46)	Tibet Plateau	31.1N | 88.7E	**3.4**	-	-
Uygur	44 (42)	Central Asia	44.6N | 87.3E	**31.1**	**50.7**	**−60.0**
Hazara	25 (23)	South Asia	34.9 N | 63.5E	**33.5**	**57.0**	**−56.6**
Pathan	25 (25)	South Asia	31.9N | 69.2E	**33.8**	**18.5**	**−21.8**
Burusho	25 (25)	South Asia	36.3N | 74.6E	**30.9**	**29.8**	**−23.8**
Makrani	25 (25)	South Asia	26.0N | 64.0E	**19.6**	−12.7	−1.8
Balochi	24 (24)	South Asia	30.5N | 66.5E	**27.9**	**2.1**	**−6.9**
Brahui	25 (25)	South Asia	30.0N | 66.0E	**26.3**	−0.6	0.4
Kalash	23 (22)	South Asia	36.0N | 71.5E	**33.2**	**14.2**	39.1
Sindhi	24 (24)	South Asia	25.5N | 69.0E	**24.3**	**8.6**	**−17.2**
French	29 (29)	Europe	48.3N | 2.6E	-	-	-
CEU	82 (82)	Europe	48.7N | 7.4E	-	**3.6**	-
GBR	89 (85)	Europe	51.8N | 0E	-	**5.0**	-
FIN	93 (93)	Europe	62.8N | 26.4E	-	**25.2**	-
Russian	25 (25)	Europe	56.2N | 37.6E	-	**24.2**	-
Adygei	17 (17)	Europe	44.6N | 40E	-	**9.4**	-
Orcadian	16 (15)	Europe	59.0 N | 3.1W	-	**4.1**	-

Note: f_4__A and f_4__B stand for Z-score of tests f_4_(YRI,French;Dai,X) and f_4_(YRI,Dai;French,X), respectively. f_3_ stands for Z-score of test f_3_(X,French,Dai). Weighted Block Jackknife (block size of 5 cM) was used to correct LD among SNPs and estimate standard deviations. For these tests, we interpreted |Z-score| ≥ 2 (bold) as significant evidence of admixture. A minus sign in table indicates that the test was not performed.

**Table 2:** Estimation of admixture proportion and admixture time

Population	Region	Gene flow (EAS%)	Date of admixture (Generation)	Date of admixture (Year)
CHB	East Asia	97.2 ± 0.2	47.7 ± 1.2	1383.3 ± 34.8
CHS	East Asia	98.3 ± 0.1	NULL	NULL
JPT	East Asia	97.8 ± 0.2	60.8 ± 31.2	1763.2 ± 904.8
Tu	East Asia	92.5 ± 0.2	43.4 ± 1.3	1258.6 ± 37.7
Hezhen	East Asia	93.2 ± 0.2	37.8 ± 7.1	1096.2 ± 205.9
Mongolian	East Asia	89.1 ± 0.1	32.9 ± 0.9	954.1 ± 26.1
Daur	East Asia	92 ± 0.2	54.5 ± 3.6	1580.5 ± 104.4
Oroqen	East Asia	90.4 ± 0.2	41.8 ± 1.6	1212.2 ± 46.4
Xibo	East Asia	89.7 ± 0.1	10.7 ± 0.7	310.3 ± 20.3
Korean	East Asia	98.4 ± 0.2	45 ± 3.9	1305 ± 113.1
Tibetan	Tibet Plateau	94.5 ± 0.4	47.8 ± 1.8	1386.2 ± 52.2
Uygur	Central Asia	47.6 ± 0.4	26.4 ± 0.5	765.6 ± 14.5
Hazara	Central Asia	49.8 ± 0.3	24.4 ± 0.2	707.6 ± 5.8
Burusho	South Asia	32.1 ± 0.3	49.2 ± 1.1	1426.8 ± 31.9
Pathan	South Asia	21.6 ± 0.3	51.2 ± 2.3	1484.8 ± 66.7
Balochi	South Asia	97.9 ± 0.4	82.0 ± 4.1	2376.9 ± 118.9
Kalash	South Asia	79.3 ± 0.3	61.8 ± 5.3	1792.8 ± 152.3
Sindhi	South Asia	89 ± 0.4	70.7 ± 3.1	2049.7 ± 89.1
CEU	Europe	0.7 ± 0.8	NULL	NULL
GBR	Europe	2.5 ± 1	82.8 ± 39.6	2401.2 ± 1148.4
FIN	Europe	12.5 ± 0.9	64.2 ± 1.1	1861.8 ± 31.9
Russian	Europe	12.4 ± 1	45.2 ± 1.3	1310.8 ± 37.7
Adygei	Europe	3.2 ± 1	24.1 ± 1.2	698.9 ± 34.8
Orcadian	Europe	3.8 ± 1	78.1 ± 8.6	2264.9 ± 249.4

Note: Admixture time was estimated with *ROLLOFF* assuming the putative ancestral populations were Dai and French. Standard errors were computed using Weighted Block Jackknife by removing one chromosome each of 22 times. We assume 29 years for each generation. NULL indicates that no obvious ALD decay was observed.
