# Supplementary material for: Quantitating and Dating Recent Gene Flow between European and East Asian Populations
Source: Sci Rep. 2015 Apr 2;5:9500. doi: 10.1038/srep09500 (PMC4382708; doi:10.1038/srep09500)
Supplement: Supplementary Information [file srep09500-s1.pdf]

Supplementary Information of

Quantitating and Dating Recent Gene Flow between European and East Asian Populations

Running title: Gene flow between Europeans and East Asians

Pengfei Qin, [1] *Ying Zhou,* [1] *Haiyi Lou,* [1] *Dongsheng Lu,* [1] *Xiong Yang,* [1] *Yuchen Wang,* [1] *Li Jin,* [2] *Yeun-Jun Chung,* [3] *Shuhua Xu,* [1,4,5] *

[1] Chinese Academy of Sciences (CAS) Key Laboratory of Computational Biology, Max-Planck Independent Research Group on Population Genomics, CAS-MPG Partner Institute for Computational Biology, Shanghai Institutes for Biological Sciences, Chinese Academy of Sciences, Shanghai 200031, China;

[2] Ministry of Education (MOE) Key Laboratory of Contemporary Anthropology, School of Life Sciences and Institutes of Biomedical Sciences, Fudan University, Shanghai 200433, China;

[3] Integrated Research Center for Genome Polymorphism, Department of Microbiology, The Catholic University Medical College, Socho-gu Seoul 137-701, Korea;

[4] School of Life Science and Technology, Shanghai Tec University, Shanghai 200031, China;

[5] Collaborative Innovation Center of Genetics and Development, Shanghai 200438, China.

* To whom correspondence should be addressed. E-mail: xushua@picb.ac.cn (S.X.)

Supplementary Figure S1 | HI model for admixture between EUR and EAS.

Admixture model for EUR and EAS, where p is the gene flow from ancestral EUR, and T and W are the quantitates of branch length or drift.

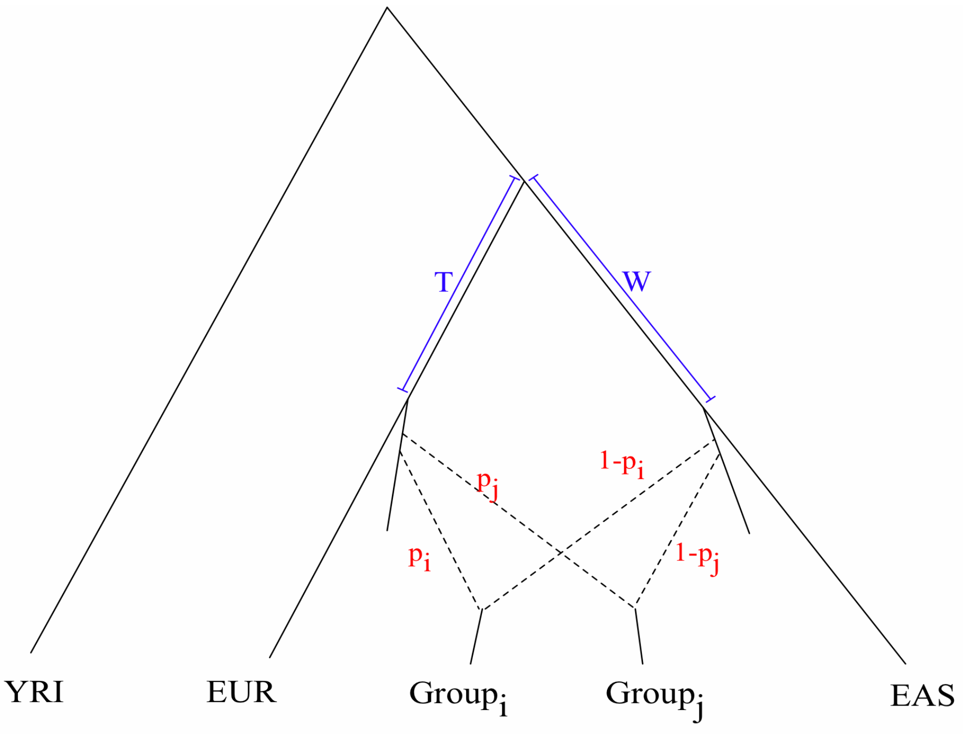

Supplementary Figure S2 | Admixture model used for estimating admixture proportions.

Papuan was used as an out-group. French and Dai were used as the surrogates of ancestral EUR and EAS, respectively.

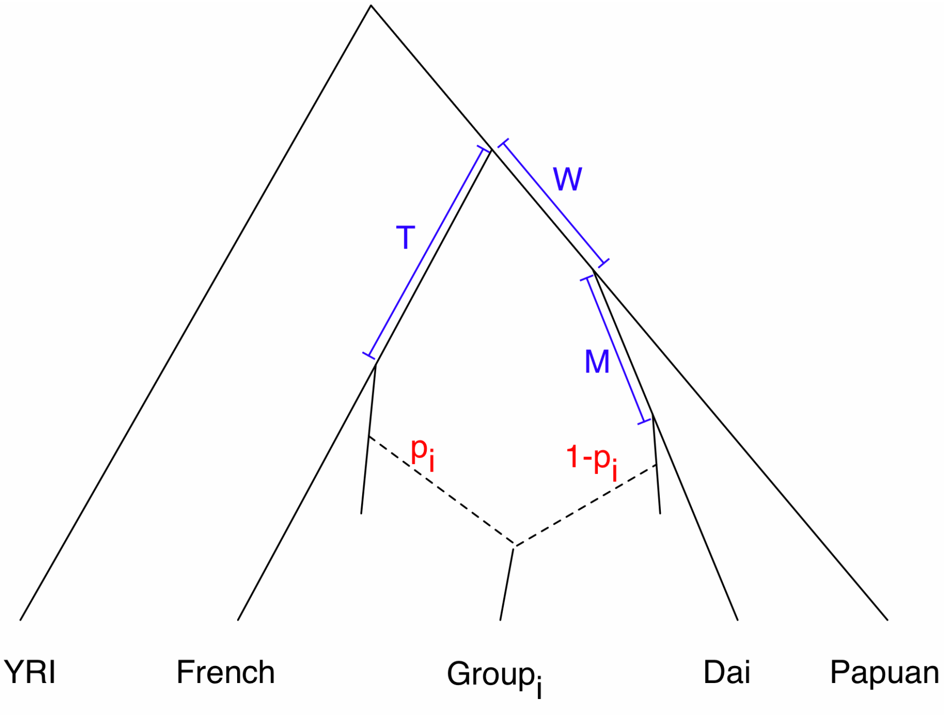

Supplementary Figure S3 | Linear regression model for ancestry estimation.

We got well-fitting linear regression models for (a) EAS, $r^2 = 0.80$ and (b) EUR, $r^2 = 0.90$ but not for (c) CSA.

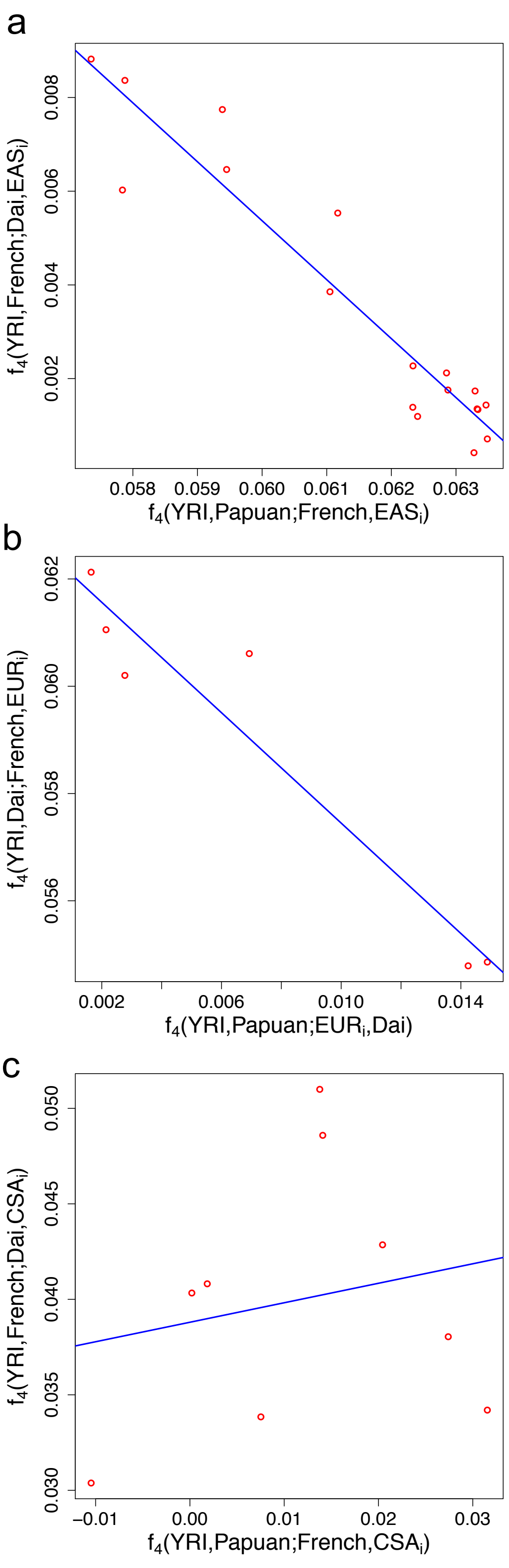

Supplementary Figure S4 | Simulations showed admixture LD of Uygur genome was influenced by recent admixture.

A1 denotes number of generations since the first (ancient) admixture event, while A2 denotes number of generations since the second (recent) admixture event. W denotes the weights of the second admixture, i.e. the proportion of ancestry contribution of the second admixture to the current admixed population.

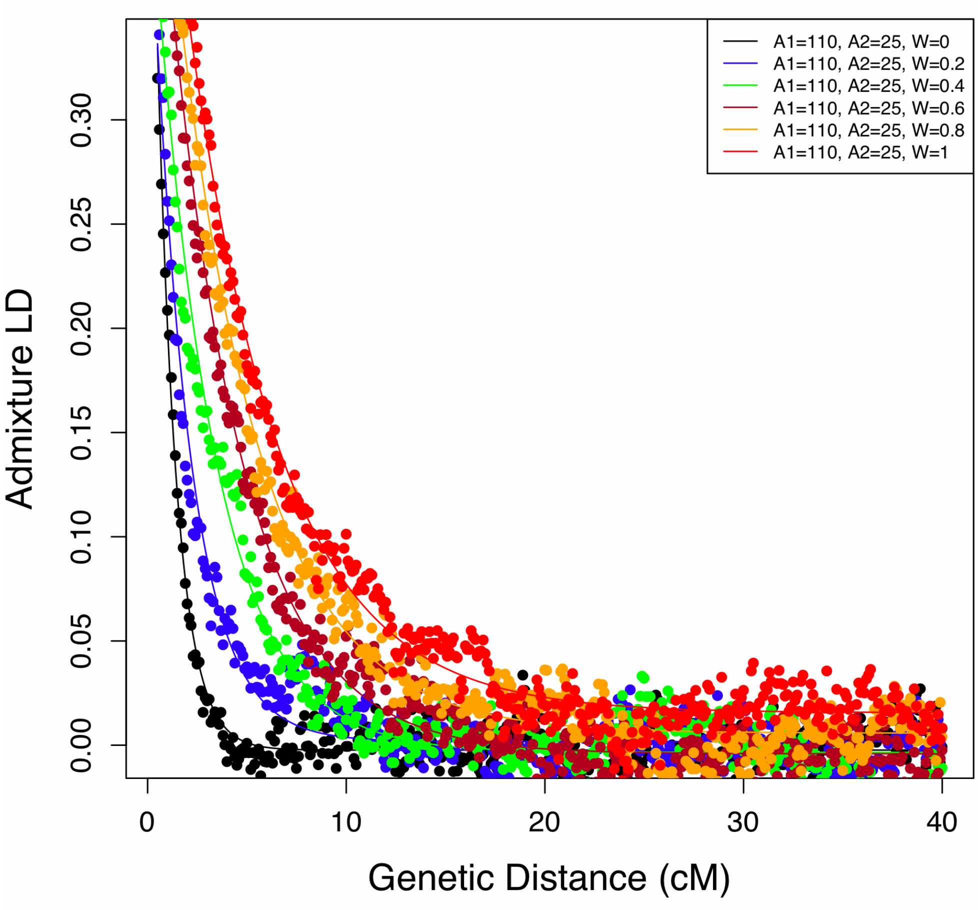

Supplementary Figure S5 | Simulation for estimation of admixture using both real ancestors and surrogates.

In the model below, population B and C are the real ancestor who directly contributed genetic material to admixed population X. Population B' and C' were the surrogates of ancestor B and C, respectively. Parameters for demographic history could be found in the command line of *ms* (basic effective population size is 10,000). Results of estimations of admixture proportion could be found in Supplementary Fig. S6.

Basic command line used to produce the simulated data by *ms* is ./ms 120 50000 -t 1 -I 6 20 20 20 20 20 20 -n 1 8 -n 2 2.5 -n 3 5 -n 4 1.5 -n 5 1.5 -n 6 2 -es 0.0015 6 0.95 -en 0.001525 7 1.0 -ej 0.0025 7 5 -ej 0.0075 6 3 -en 0.007525 3 0.33 -ej 0.01 5 2 -en 0.010025 2 0.7 -ej 0.025 4 3 -ej 0.03 3 2 -en 0.030025 2 0.25 -ej 0.06 2 1 -en 0.060025 1 1

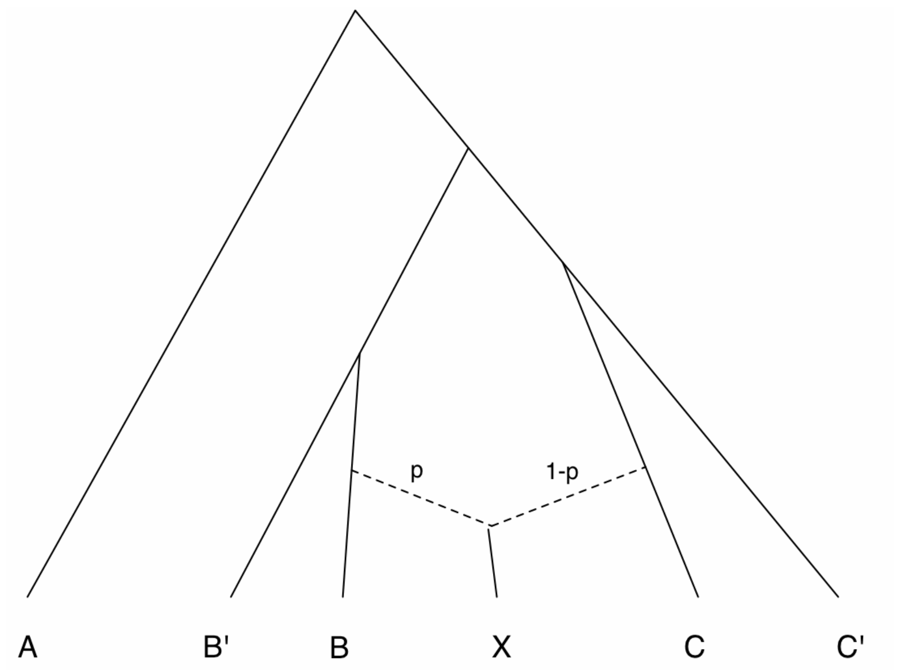

Supplementary Figure S6 | Simulation studies showed surrogate of ancestor and ascertainment bias will not affect estimates of admixture proportion. (a) Using surrogates instead of real ancestors would not affect the estimations of admixture proportion. (b) Ascertainment bias would not affect the estimations of admixture proportion. We simulated ascertainment bias by using markers with minor allele frequency (MAF of all populations, EUR only and EAS only) > 5%

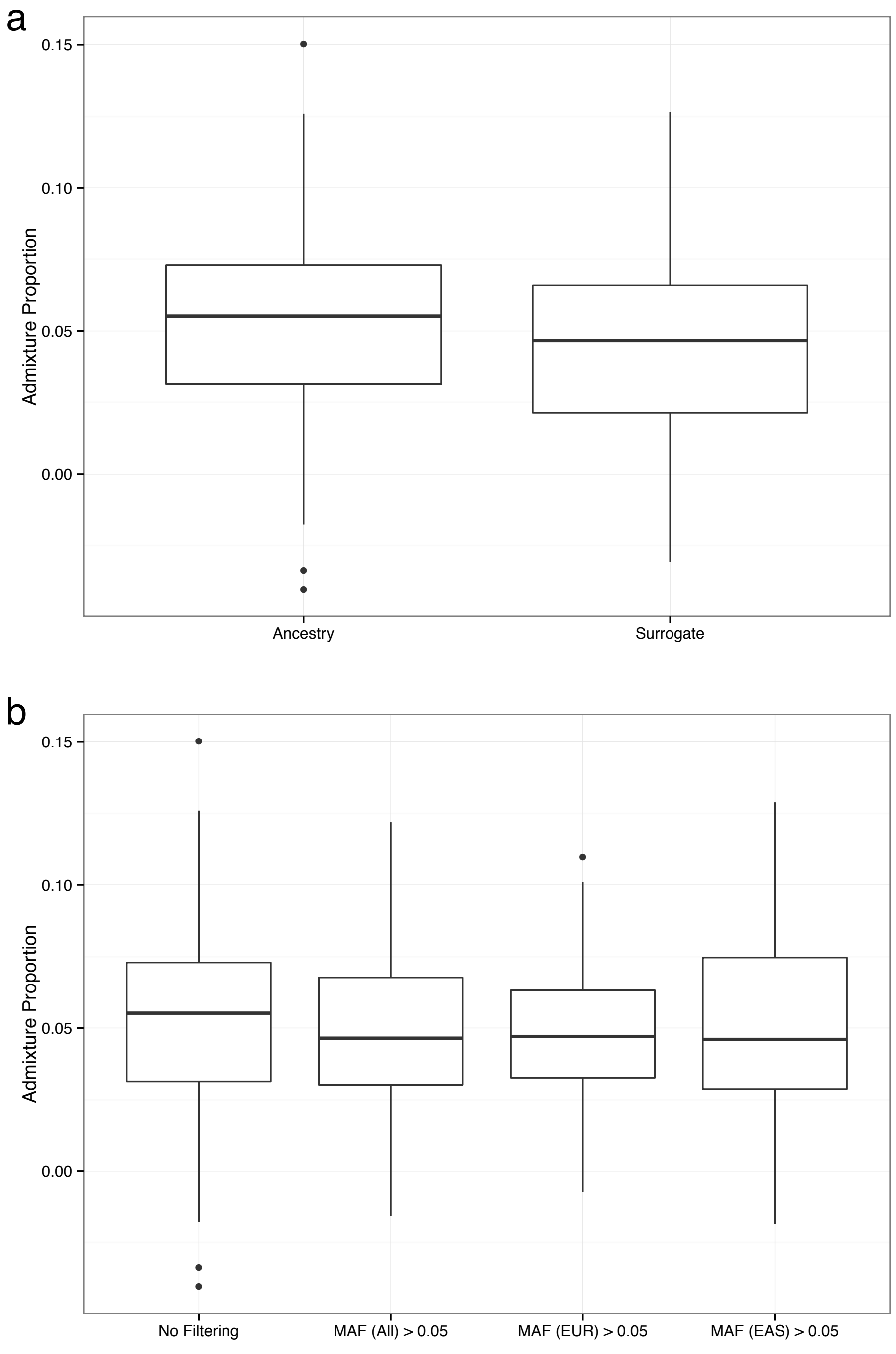

Supplementary Figure S7 | Simulation for estimation of admixture proportion when the real ancestor is pre-mixed.

In the model below, population B and C are the real ancestor who directly contributed 50% genetic material to admixed population X. Population C is pre-mixed who inherited 5% genetic material from D. Parameters for demographic history could be found in the command line of *ms* (basic effective population size is 10,000). Results of estimations could be found in Supplementary Fig. S8.

Basic command line used to produce the simulated data by *ms* is

./ms 100 50000 -t 1 -I 5 20 20 20 20 20 -n 1 8 -n 2 2.5 -n 3 5 -n 4 5 -n 5 5 -es 0.000625 5 0.5 -en 0.00065 6 1.0 -ej 0.000675 6 4 -es 0.00075 4 0.95 -en 0.000775 7 1.0 -ej 0.0008 7 2 -en 0.020025 3 0.33 -en 0.020025 2 0.7 -ej 0.03 3 2 -en 0.030025 2 0.25 -ej 0.06 2 1 -en 0.060025 1 1

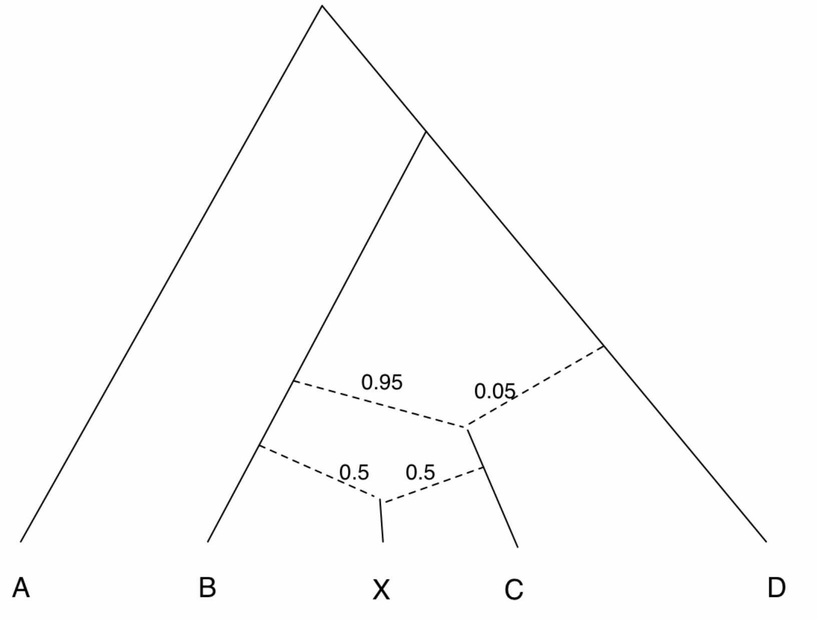

Supplementary Figure S8 | Estimates of admixture proportion in simulations when the real ancestor is pre-mixed.

In simulations (based on the model in Supplementary Fig. S7), we considered both full markers (red box) and markers with minor allele frequency > 5% (blue box). One ancestor is pre-mixed who inherited 5% genetic material from European and contributed 50% to the new admixed population (expected admixture proportion for the new admixed population is 2.5%). Admixture proportion was re-estimated by F_4 ratio based on simulated data. Results showed neither ascertainment bias nor pre-mixed ancestor would affect the estimations of admixture proportion.

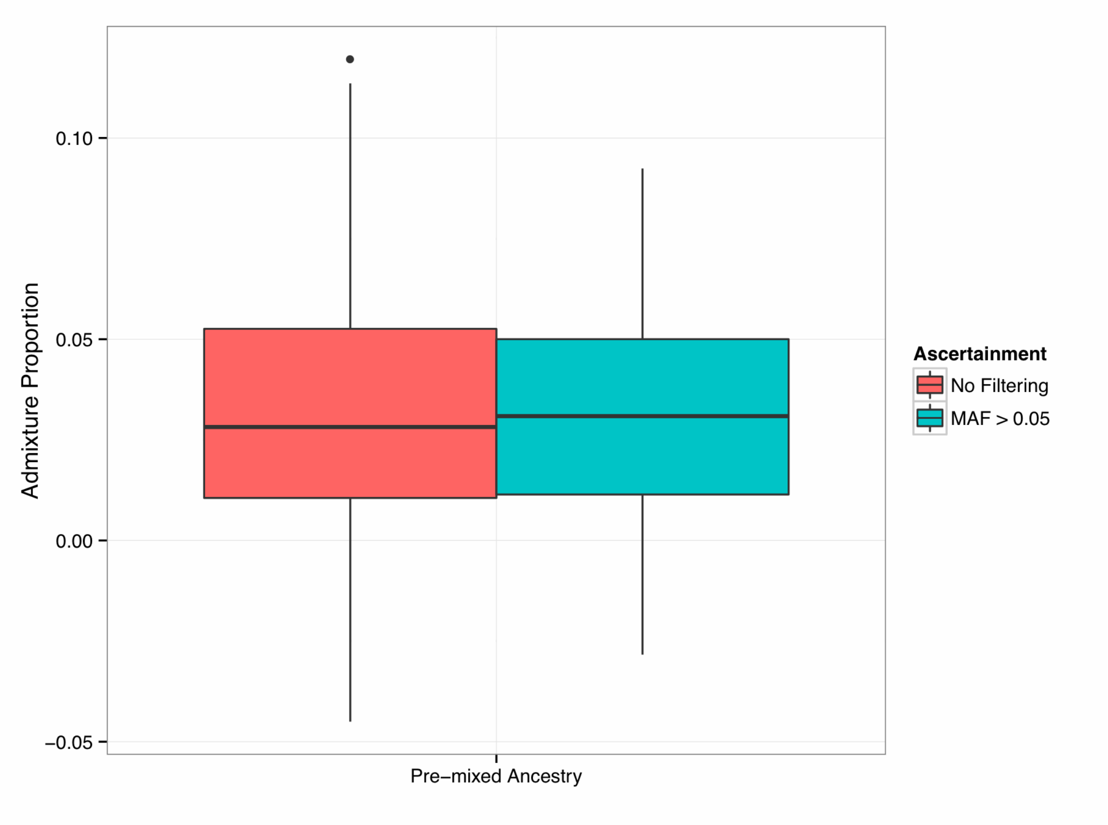

Supplementary Figure S9 | Simulation and dating admixture when the real ancestor is pre-mixed.

(a) Admixture scenario for simulations (Similar to the history of Xibo). Ancestor C was pre-mixed at 110 generations ago. Population X was admixed by A and C at 10 generations ago. (b) Admixture time was estimated by *ROLLOFF*. Simulation and dating were repeated with 50 times.

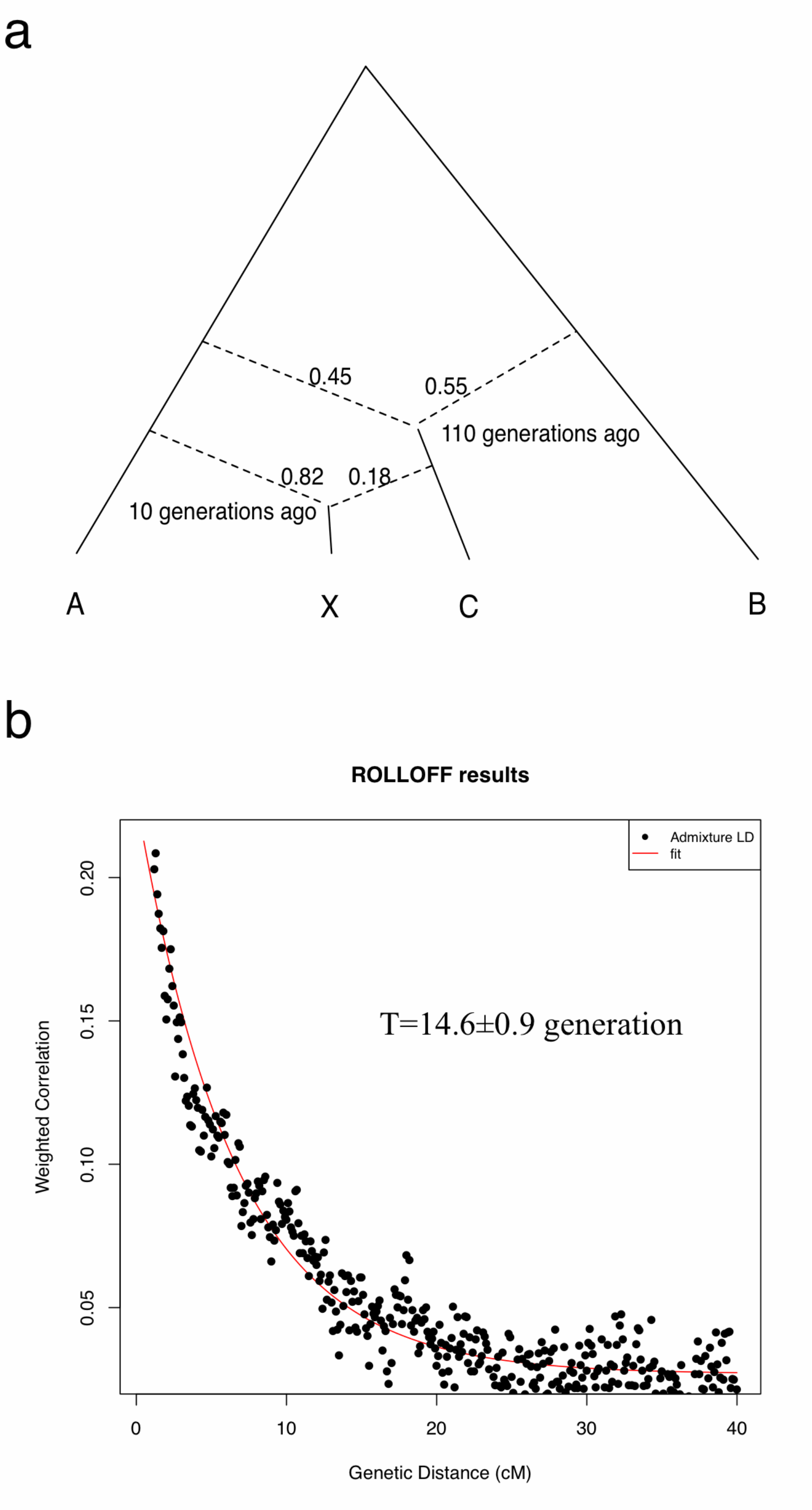

Supplementary Figure S10 | Definition of haplotype segments we used to estimate admixture time.

Number of segments with one allele from ancestry A and the other allele from ancestry B were used to estimate expected admixture time based on Equation 5.

Blue bars are the segments from ancestry A, red bars are segments from ancestry B. S is the segment we count.

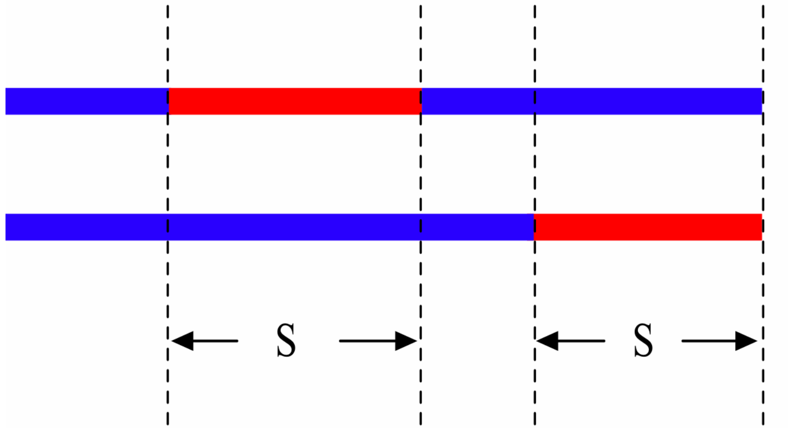

Supplementary Table S1 | Testing for gene flow between EUR and EAS.

Population	Region	f_4_A		f_4_B		f_3	
		Value	Zscore	Value	Zscore	Value	Zscore
Dai	East Asia	0.0000	-	-	-	-	-
CHB	East Asia	0.0023	**3.7**	-	-	-	-
CHS	East Asia	0.0013	**2.3**	-	-	-	-
JPT	East Asia	0.0018	**2.6**	-	-	-	-
Lahu	East Asia	0.0004	0.5	-	-	-	-
Naxi	East Asia	0.0012	1.4	-	-	-	-
She	East Asia	0.0007	0.9	-	-	-	-
Yi	East Asia	0.0014	1.8	-	-	-	-
Miao	East Asia	0.0014	1.9	-	-	-	-
Tujia	East Asia	0.0014	1.8	-	-	-	-
Tu	East Asia	0.0060	**7.6**	-	-	-	-
Hezhen	East Asia	0.0055	**6.2**	-	-	-	-
Mongolian	East Asia	0.0088	**10.3**	-	-	-	-
Daur	East Asia	0.0065	**7.3**	-	-	-	-
Oroqen	East Asia	0.0077	**8.6**	-	-	-	-
Xibo	East Asia	0.0084	**9.85**	-	-	-	-
Korean	East Asia	0.0018	**2.55**	-	-	-	-
Tibean	Tibet Plateau	0.0020	**3.4**	-	-	-	-
Uygur	Central Asia	0.0351	**31.1**	0.0717	**50.7**	-0.0461	**-60**
Hazara	Central Asia	0.0346	**33.5**	0.0676	**57**	-0.0411	**-56.6**
Pathan	South Asia	0.0487	**33.8**	0.0162	**18.5**	-0.0149	**-21.8**
Burusho	South Asia	0.0429	**30.9**	0.0294	**29.8**	-0.0166	**-23.8**
Makrani	South Asia	0.0305	**19.6**	-0.0117	-12.8	-0.0013	-1.8
Balochi	South Asia	0.0410	**27.9**	0.0019	**2.1**	-0.0049	**-6.9**
Brahui	South Asia	0.0406	**26.3**	-0.0005	-0.6	0.0003	0.4
Kalash	South Asia	0.0515	**33.2**	0.0147	**14.2**	0.0440	39.1
Sindhi	South Asia	0.0339	**24.3**	0.0078	**8.6**	-0.0120	**-17.2**
French	Europe	-	-	-	-	-	-
CEU	Europe	-	-	0.0016	**3.6**	-	-
GBR	Europe	-	-	0.0021	**5**	-	-
FIN	Europe	-	-	0.0142	**25.2**	-	-
Russian	Europe	-	-	0.0149	**24.2**	-	-
Adygei	Europe	-	-	0.0069	**9.4**	-	-
Orcadian	Europe	-	-	0.0028	**4.1**	-	-

Note: f_4_A and f_4_B stand for tests f_4(YRI,French;Dai,X) and f_4(YRI,Dai;French,X), respectively. f_3 stands for test f_3(X,French,Dai). Weighted Block Jackknife (block size of 5 cM) was used to correct LD among SNPs and estimate standard deviations. For these tests, we interpreted |Z-score| ≥ 2 (bold) as significant evidence of admixture. A minus sign in table indicates that the test was not performed.

Supplementary Table S2 | Estimation of admixture level using two methods

Population	Region	Gene Flow (regression) EAS : EUR (SE)	Gene Flow (f_4 ratio) EAS : EUR (SE)
CHB	East Asia	97.2: 2.8 (0.2)	98.2: 1.8 (0.3)
CHS	East Asia	98.3: 1.7 (0.1)	99.7: 0.3 (0.3)
JPT	East Asia	97.8: 2.2 (0.2)	99.0: 1.0 (0.3)
Lahu	East Asia	99.5: 0.5 (0.2)	99.7: 0.3 (0.3)
Naxi	East Asia	98.5: 1.5 (0.2)	98.3: 1.7 (0.3)
She	East Asia	99.1: 0.9 (0.2)	100.0: 0.0 (0.3)
Yi	East Asia	98.2: 1.8 (0.2)	99.9: 0.1 (0.3)
Miao	East Asia	98.3: 1.7 (0.2)	99.7: 0.3 (0.3)
Tujia	East Asia	98.3: 1.7 (0.2)	98.2: 1.8 (0.3)
Tu	East Asia	92.5: 7.5 (0.2)	91.1: 8.9 (0.3)
Hezhen	East Asia	93.2: 6.8 (0.2)	96.3: 3.7 (0.3)
Mongolian	East Asia	89.1: 10.9 (0.1)	90.3: 9.7 (0.3)
Daur	East Asia	92.0: 8.0 (0.2)	93.6: 6.4 (0.4)
Oroqen	East Asia	90.4: 9.6 (0.2)	93.5: 6.5 (0.3)
Xibo	East Asia	89.7: 10.3 (0.1)	91.1: 8.9 (0.3)
Korean	East Asia	98.4: 1.6 (0.2)	98.1: 1.9 (0.3)
Tibetan	Tibetan Plateau	-	94.5: 5.5 (0.4)
Uygur	Central Asia	-	47.6: 52.4 (0.4)
Hazara	Central Asia	-	49.8: 50.2 (0.3)
Burusho	South Asia	-	32.1: 67.9 (0.3)
Pathan	South Asia	-	21.6: 78.4 (0.3)
Balochi	South Asia	-	97.9: 2.1 (0.4)
Kalash	South Asia	-	79.3: 20.7 (0.3)
Sindhi	South Asia	-	89: 11 (0.4)
CEU	Europe	0.7: 99.3 (0.8)	2.2: 97.8 (0.1)
GBR	Europe	2.5: 97.5 (1.0)	3.9: 96.1 (0.1)
FIN	Europe	12.5: 87.5 (0.9)	13.7: 86.3 (0.2)
Russian	Europe	12.4: 87.6 (1.0)	13.6: 86.4 (0.2)
Adygei	Europe	3.2: 96.8 (1.0)	4.6: 95.4 (0.2)
Orcadian	Europe	3.8: 96.2 (1.0)	5.2: 94.8 (0.2)

Note: Two methods (Regression Ancestry Estimation and F_4 Ratio Estimation) were used to estimate the proportions of admixture. Tibetan and CSA were not included in the regression analysis because Tibetan has quite different migration history and geographical location (a minus sign in the table).

Supplementary Table S3 | Simulation of admixture of the Uygur assuming two pulses of admixture and estimation of admixture time based on empirical and simulated data.

	Admix 1	Admix 2	W2	ALD-based	Segments-based
**Empirical**	NA	NA	NA	26.4 ± 0.5	54.2 ± 1.1
**Simulated**	110	25	0	106.6 ± 5.9	104.6 ± 1.8
	110	25	0.2	57.7 ± 7.5	88.8 ± 0.4
	110	25	0.4	32.6 ± 3.7	71.7 ± 1.8
	110	25	0.6	27.3 ± 3.7	57.6 ± 1.7
	110	25	0.8	25.5 ± 0.4	39.8 ± 0.9
	110	25	1.0	22.2 ± 1.6	23.8 ± 0.6

Note: Admix 1 and Admix 2 represent number of generations for the first (ancient) and the second (recent) admixture event, respectively. W2: weight of admix 2, which denotes the proportion of ancestry contributed by the second admixture event, consequently, ancestry contribution of the first admixture event to the current admixed population (W1) is (1- W2). NA: unknown or not available.
